# Association between Attitude and Empathy with the Quality of Human-Livestock Interactions

**DOI:** 10.3390/ani10081304

**Published:** 2020-07-30

**Authors:** Andres Felipe Leon, Jorge Alberto Sanchez, Marlyn H. Romero

**Affiliations:** 1 Faculty of Agrarian and Animal Sciences, University of Caldas, Manizales 170004, Colombia; andres.1992.leon@gmail.com; 2 Department of Animal Health, Faculty of Agrarian and Animal Sciences, University of Caldas, Manizales 170004, Colombia; jorge.sanchez@ucaldas.edu.co

**Keywords:** human-animal relationship, livestock market, wellbeing

## Abstract

**Simple Summary:**

The attitudes and empathy of handlers influence human–animal interaction, thereby affecting their behavior toward animals. In this study, we evaluated the association between the attitude and empathy of livestock handlers towards the animals with the quality of human-livestock interactions. A structured survey instrument was used to assess managers’ attitudes and empathy towards livestock. The quality of handling was assessed by recording the individual behavior of each handler during commercial handling procedures; furthermore, it was ascertained whether the quality of handling was affected throughout the workday. Associations were found between the quality of the interactions used by the handlers towards the animals and the positive or negative attitude and empathy scores. It has been concluded that there is an association between handlers’ attitudes and empathy towards animals and the quality of human-animal interaction pre-slaughter.

**Abstract:**

The human-animal interactions are a key component of human and animal welfare. The quality of this interaction can therefore be assessed by measuring the reaction response of the animals to the handler’s behavior. The aim of this study was to investigate the association between attitude and empathy towards the animals with the quality of human-livestock interactions. Additionally, we aimed to investigate whether the quality of cattle handling deteriorates as the working day progresses. A total of 18 livestock handlers and 1514 Colombian commercial Zebu steers were evaluated. A questionnaire pack consisting of 50 questions regarding demographic information, attitude and empathy characteristics was applied, using a structured interview. Each handlers’ responses to positive and negative attitude and empathy questions were calculated to produce a composite score. Observations of human-animal interactions were made at three times during the day (5:00, 7:00 and 9:00), each observation lasting 30 min. The handlers had an average age of 39.4 ± 3.4 y (range = 18–66 y), with little schooling but a lot of experience in the trade (17.13 ± 14.21 y). During handling, hitting, prodding and hand raising predominated over other actions (*p* < 0.05), and in response, the cattle behaved by freezing and running. Significant differences were found in the interactions used by handlers depending on the time of day (*p* < 0.05). The empathy total score ranged from 20 to 100, and the attitude total score between 24 and 120. The average attitude and empathy scores for handlers were 85.05 ± 6.92 (mean ±SD; range, 73–97) and 74.61 ± 4.72 (mean ±SD; range, 65–83), respectively. It has been concluded that there is an association between handlers’ attitudes and empathy towards animals and the quality of human-animal interaction during pre-slaughter.

## 1. Introduction

In recent years, there has been an increased interest in animal welfare, both in scientific research and in society, with the aim to minimize animal suffering and to promote their positive emotions and welfare [1]. Pre-slaughter is a complex stage in the cattle logistics chain, because of the implications for animal welfare, transport losses, occupational risk and meat quality, among other factors [2]. One of the factors that has the greatest impact on animal welfare during pre-slaughter management is the quality of the interactions between the animals and their handlers [3]. They affect work comfort and efficiency, as well as meat quality. A poor human-animal relationship can result in stress and accidents to both animals and handlers and needs to be improved [4]. Likewise, handlers are of vital interest, not only from an animal welfare perspective but also for the One-Welfare concept. One- Welfare concept shows how animal welfare is interconnected with human wellbeing, biodiversity and the environment at different levels of society [5]. In the livestock industry, One-Welfare could help to promote key global objectives, such as standards that guarantee the health and welfare of farm animals, preventing or reducing occupational hazards that may affect handlers, promote sustainability in animal production and generate an integrative vision of human-animal interactions [6].

Empathy plays an important role in interpersonal relationships, and it shapes the relationships between human and nonhuman species, affecting the way that animals are treated and cared for [7]. However, empathy in livestock handlers has received very little attention so far. Likewise, it is well known that livestock handlers’ attitudes towards their animals is directly associated with their behavior during handling and that rough practices can negatively affect animal welfare and increase animals’ fear towards humans [8].

Livestock markets can be defined as specific locations with dedicated facilities where buyers and sellers come together to trade live animals [9]. The livestock market environment is likely to contain a number of fear provoking stimuli for cattle, including novel and startling stimuli, intense stimuli such as loud noises, evolutionarily relevant stimuli such as their height above ground, isolation, and darkness, auditory and olfactory alarm signals from conspecifics, additional periods of food and water restriction and aversive handling [10]. Likewise, the activities of livestock markets require handlers to be in constant contact with the animals, and it has been shown that animals may face aversive handling during daily work routines [11].

In Colombia, there are some livestock industry initiatives to promote animal welfare and handling practices in the livestock markets. In 2010, with the support of an academy and other state institutions, a program called “Professional livestock handlers” was implemented for livestock handlers working at an auction/livestock market in Medellin. Its objective is to train livestock handlers in an integral way using social, family and personal aspects; as well as in the practices of cattle handling. However, limited research has been done in Latin America assessing whether a change in handlers’ attitudes and empathy towards animals can improve human-animal interaction during the daily work routine. It has been reported that animal handling becomes more difficult and increases stress levels when handlers do not know how to properly handle the animals, an aspect that can affect the behavior of handlers during the daily work routine [8]. It has been suggested that fatigue can negatively affect handlers’ attitudes and empathy towards animals, and under these conditions, handlers will tend to be abusive towards the animals [8]. Grandin [2] suggests that injuries and deaths of birds and pigs in commercial production can double during truck loading when handling staff time is longer than 6 h.

Although there are numerous studies in the world that have evaluated the association between the empathy and attitude of handlers towards animals, with the quality of their management practices [3], as well as the response of animals to different human interactions in productive systems and pre-slaughter [10]—it is interesting to establish whether the management conditions and practices traditionally used by handlers in Latin America are having the same implications in livestock management. Knowledge of these aspects can help to strengthen training programs for livestock handlers and the implementation of sanitary legislation, which was recently updated with a comprehensive farm-to-table approach, as is the case in Colombia [12]. The aim of this study was to investigate the association between the attitude and empathy of livestock handlers with the quality of human-livestock interactions; the quality of this interaction was assessed by measuring the reaction response of the animals to the handler’s behavior. Additionally, we aimed to investigate whether the quality of cattle handling changes as the working day progresses. We hypothesized that livestock handlers with good attitudes and empathy levels towards animals would have better interactions and more positive management behaviors with the cattle, and that this management would be consistent throughout the workday.

## 2. Materials and Methods

The study was carried out in the department of Antioquia (northwestern Colombia) from August to October 2018, at a livestock market in Medellin (6°13′00″ N 75°34′00″ W). Medellin is characterized by having a tropical rain forest climate with a mean annual rainfall of 2.550 mm and mean annual temperature of 16.6 °C.

### 2.1. Ethical Note

The study was carried out under commercial livestock market conditions, and the researchers participated in the process solely as observers. All procedures related to the use and care of the animals strictly followed the Colombian regulation norm, Resolution 001634-2010, as stated by the Colombian Agricultural Institute [13]. Permission to conduct the study was approved by the Ethics Committee for Animal Experimentation (Act 24/06/2018, Activities with minimal risk) and the Human Ethics Committee (Act 15/06/2018) at the University of Caldas. Livestock handlers were fully debriefed about the purpose of the study, and they read/listened and signed an informed consent form and authorization to allow us to use the data.

### 2.2. Studied Livestock Market and Handlers

**Figure 1:**
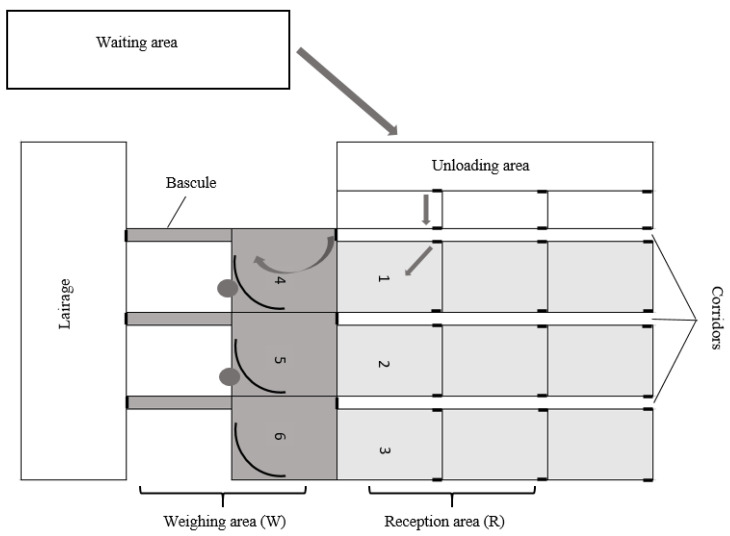
Schematic representation of observed sections of the driving areas from the corridors to the reception area (light grey and white), and from the reception area to the weighing area (dark grey).

The livestock market is part of a livestock center that provides trading services for cattle, pigs and horses; it also has auctions for breeding cattle and commercial livestock, and is used for slaughtering cattle and pigs. The livestock market traded 5000 cattle per week. The livestock market had 16 unloading ramps with non-slip and cement floors. They were connected by a series of corridors (23.7 m wide × 5.7 m long) to a reception area that consisted of 22 pens (5.9 m wide × 9.8 m long; 57.8 m^2^), with nonslip concrete floors. A concrete passageway guided animals from the reception area (R) to a weighing area (W) (Figure 1).

All animals arrived at the livestock market on Saturday; they were unloaded from the transport vehicle on Sunday and kept in outdoor lairage overnight. Animals from different livestock trucks were mixed. The animals that entered the livestock market on Sunday were slaughtered on Monday, according to the schedule of the slaughterhouse; the calves that entered on Monday were marketed for slaughter once they complied with the fasting time required by health regulations, or to enter farms as replacement animals. Animal handling started on Monday at 05:00 h. Record keeping of behaviors and interactions were made at two different moments of the handling process: in the last part of the driving area from the corridors to the reception area, and from the reception area to the weighing area. An average of 30 handlers participated in the process, of which 18 agreed to participate in the study, they aged between 18 and 66 years, and the length of service ranged between 2 and 50 years. Similarly, data were recorded from 1514 Colombian commercial Zebu steers (Brahman, Guzerat, and Nellore crossbreds), that ranged from 18 to 24 months of age, with an average live weight of 442.5 ± 56.8 kg. These commercial phenotypes are very common in extensive grazing systems; approximately 70% of slaughtered cattle come from this genetic background in Colombia [12].

### 2.3. The Handlers Empathy and Attitudes Questionnaire

An ad hoc questionnaire (Table A1) was applied using a structured interview [14,15,16]. The questionnaire was adapted into local Spanish by two bilingual people using the back-translation method [17]. First, a bilingual person translated the English questionnaires into Spanish. Second, another bilingual person retranslated (back translated) the Spanish adaptations to English. Finally, the original source and back translated items were compared for equivalence of meaning, and any discrepancies were noted. This reiterative process of translation and backtranslation was continued until no semantic differences were noticed between the questionnaire forms. A pilot study was carried out in June 2018 using a draft questionnaire and it was applied to 8 handlers (these participants were excluded from a subsequent questionnaire), then the results were used for the development of the final questionnaire that contained 50 questions divided into three sections. Part 1 aimed at obtaining information on the participants’ age, marital status, number of children, length of service, level of schooling, animal welfare training, knowledge of the balance point and flight zone concepts [18], as well as two questions about how to handle cattle. Part 2 (24 items) focused on the handlers’ attitudes toward cattle and handling practices (adapted from Boivin et al. and Hanna et al. [14,15]). The last part (also with 20 items) evaluated the empathy of the handlers towards the animals [16]. The original scales of attitude and empathy demonstrated concurrent validity, with correlations ranging from 0.83 to 0.92 and acceptable internal consistency with Cronbach’s alpha ranging from 0.77 to 0.86 [14,15]. Empathy and attitude responses were gathered on a five-point Likert scale, ranging from ‘strongly agree’ to ‘strongly disagree’ [15]. The negatively framed items were reversed for the purpose of analysis. Each handler’s responses to positive and negative attitude and empathy questions were calculated to produce a composite score (adapted from Breuer et al. [19]). The empathy total score ranged from 20 to 100, and the attitude total score between 24 and 120; with higher scores indicating higher levels of self-reported perception of empathy and attitude toward cattle (adapted from Colombo et al. [7]).

In order to reduce the influence of the observers on the handlers’ behavior, the detailed objective of the study was not revealed to the participants until the study was completed. The researchers who conducted the surveys were at the livestock market for four weeks observing the work, gaining the participants’ trust and getting them used to their presence during the daily routines. This meant that the participants were partially aware of the purpose of the study.

The interviews were conducted one month after assessing the quality of cattle handling. As not all handlers were able to read the questionnaire, interviewers read each question without any comment for all respondents, and recorded their responses in categories from 1 (complete disagreement) to 5 (complete agreement). The handlers who participated in the research were at liberty to remain in the study and in the interviews, and were informed of this prior to being interviewed. At the end of the study, all participants were informed of the full purpose of the study, and it was made known that the information obtained would be handled anonymously during the analysis, interpretation and publication of the results.

### 2.4. Assessing Livestock Handlers and Animal Behavior During Handling

The quality of handling was assessed by recording the behaviors of each handler and animal, individually, during handling procedures. Observations were made at three times of the day (5:00, 7:00 and 9:00), and each observation lasted for 30 min. Cattle evaluated at 5:00 h arrived on Saturday at the livestock market; steers evaluated at 7:00 and 9:00 h arrived on Sunday night and Monday morning, respectively. Animals and the livestock handlers were observed simultaneously using continuous observations. Each livestock handler included in the study was evaluated individually. A focal selection of animals was made (group of 14 to 15 steers in each evaluation) using the double-blind method, where neither the handlers nor the observers knew the day of entry, nor the origin of the steers. At the time of the human-animal interaction evaluation, only the lot number was recorded, and once this process was finished, the observers searched the cattle market database for information related to the date of entry and lairage time of the animals evaluated. At each observation session, two observers were used (always the same people) simultaneously; one observed the focal animals and the other researcher observed the livestock handlers. Before starting observations, the observers were trained in a pilot test using video clips on a desktop computer, but it was not possible to estimate the level of agreement between and within different observers. The behavioral events recorded were falls (when an animal dropped down from a higher level to a lower level), aggression/fight (antagonistic behavior observed among animals), slips (when an animal lost its balance temporarily), jumps (when an animal passed over something by jumping), baulks (when an animal stopped suddenly and refused to walk for 10 s), reversing (when an animal moved backwards), freezing (when an animal stops a forward movement and does not start to walk, despite contact or encouragement by the handler), running (moving at any pace faster than a walk) and vocalization (bellow, moo) [20].

The frequencies of interactions were recorded as tactile, auditory and visual. Tactile interactions of humans included hitting (when the handler hits with the hand or an object), prodding (when the handler prods with an object such as a wooden stick) and tail twisting (when the handler twists the tail to move the steer). Auditory interactions included talking, shouting, whistling and the use of artificial noises, such as the banging of pen fittings. Waving (the movement of objects or hands in front of the animals), blocking (when the handler stands in front of the animal and raises his hands to prevent it from moving) and the raising of hands were the visual interactions recorded [21,22]. Human-animal observations were made within regular working hours.

### 2.5. Statistics

The software Stata Version 12.0 (College Station, Texas, EU) was used for all the statistical analyses. Firstly, a normality test of the evaluated variables was carried out, and the variables with non-normal distributions were transformed by means of the natural logarithm and square root, and these values were used for later statistical analysis. A descriptive statistic was used to describe the frequency of interactions of the handlers and the behavior response of the cattle during handling; this is presented in their natural scale means ±SD. Considering that the lairage time of the steers in the livestock market was different, a univariate statistical analysis was made to establish if it was associated with the behavioral response of the animals during the handling. Poisson regression was used in order to establish the differences in human-animal interaction according to the timetable being evaluated (5:00, 7:00 and 9:00 h); where the group was considered as an experimental unit. In the Poisson regression models, the duration of each drive (min) was included as a covariate, in order to standardize the observations. A relative risk (RR) that is greater (smaller) than 1 indicates that the variable is more (less) likely to be present in a specific category of the predictor variable compared to the reference category. Pearson correlation (r) was used to measure the degree of relationship between the interactions of handlers (per min) and the number of different cattle behaviors (per min); correlations were classified as weak (r = 0.26–0.5), moderate (r = 0.51–0.75) and strong (r = 0.76–1.0). The Attitude and empathy questionnaires had an acceptable consistency with Cronbach’s alpha (0.73 and 0.70). We applied a multivariate factor analysis to the questionnaire data to measure attitude and empathy scales independently. To accomplish this, we extracted main factors through three principal component analyses (PCA) with varimax rotation. Data input for the PCA was determined by calculating all the answers to each question for attitude and empathy separately (number of answers ranging from 1 to 5 per question). Factor analyses of the 44 items generated three components per PCA. These components contained ten and six questions of attitude and empathy, respectively, with loadings above 0.5 (Table 1 and Table 2). Based on the factor analysis results, each handler’s responses were summed up to produce composite scores for the three positive and ten negative attitude questions and for the four positive and two negative empathy questions. In this process, we obtained two composite scores to describe attitude and empathy scores (adapted from Ceballos et al. [8]); to evaluate the effects of the handler’s attitude and empathy (positive and negative) on the quality and behavior of handling, simple linear regressions were used, by means of the adjusted coefficient of determination R^2^. A probability level of *p* < 0.05 was chosen as the limit for statistical significance in all tests, whereas probability levels of *p* ≤ 0.10 were considered as a tendency.

## 3. Results

### 3.1. Sociodemographic Characteristics and Handling Concepts

The livestock handlers had an average age of 39.44 ± 3.43 years (range = 18–68 years), with experience in the trade (17.13 ± 14.21 y, range = 1.5–50 years). Notably, 33.33% (*n* = 6) of the handlers had no children. In terms of schooling, 44.44% (*n* = 8) had studied at primary level, 33.33% (*n* = 6) at secondary level, and 22.22% (*n* = 4) were illiterate. Moreover, 83.33% (*n* = 15) were familiar with the concepts of the flight zone and the point of balance for livestock. Furthermore, to the question “do you know what the protocol is in the market to handle non-ambulatory disabled cattle, that is cattle that cannot rise from a recumbent position or cannot walk, including, but not limited to, those with broken appendages, severed tendons or ligaments, nerve paralysis, fractured vertebral column or metabolic conditions”, 77.78% (*n* = 14) answered in the affirmative.

The main motivation for practicing their trade was necessity (77.79%) and enjoyment (22.21%). As to the question “Do you believe in handling the animals in such a way that they are less stressed during their stay at the livestock market?”, 33.33% answered in the affirmative, and 55.56% (*n* = 10) said that their mood was reflected in the way that they treated the cattle during their daily routine.

### 3.2. Handling Assessment

Handling time averages at both moments were evaluated significantly differently (1.11 ± 0.02 (R) versus 2.73 ± 0.08 (W) min/batch); therefore, the interactions used by the staff and the behavioral response of the steers were adjusted by the duration of each handling evaluated. Table 2 shows the adjusted means and deviation standard (±SD) of the interactions used by handlers (per min) and the behavioral events of animals (per min) observed during this handling. The interactions employed the most were hitting, prodding, shouting, raising hands and whistling. At the livestock market, electric prods were not used; this is because flag use had been established for livestock handling. However, two practices were observed with low frequency that are considered prohibited by the livestock market administration, a practice which consisted of introducing a stick into the rectum of the animals (2.32%) and twisting the tail (1.61%). The most frequent behavioral responses of the steers were freezing (72.03), running (12.43%) and reversals (9.87%) (Table 3).

Table 4 presents Pearson’s correlation coefficients of the interactions used by staff and the behavioral responses of steers during handling in the cattle market. The behavioral response of freezing presented a statistically significant correlation with hitting, prodding, shouting, whistling and the use of artificial noises.

### 3.3. Effect of the Time Elapsed within a Workday on the Handling and Behavior Reactions

Significant differences were found in the interactions used by handlers according to the time elapsed within a working day. Greater tactile interactions (hitting, prodding, tail twisting) and the use of artificial sounds were observed at the beginning of the day (05:00 h), compared to the 09:00 h time (RR < 1; *p* < 0.05). Similarly, the steers’ behavioral events (reversals and freezing) were lower during the 9:00 h evaluation, compared to the 05:00 h time (RR < 1; *p* < 0.05) (Table 4). In contrast, tail twisting presented a higher frequency at 9:00 am. Staff interactions and behavioral responses of steers that did not present significant differences according to the time schedule being evaluated were not included in Table 5.

### 3.4. Assessment of the Levels of Attitude and Empathy

Principal component analysis for attitude identified three main factors with eigenvalues greater than 1 (2.7, 2.1, and 1.7); they represented 53% of variance, Factor 1 represented 22% of the variance and differentiate types of interactions with cattle; Factor 2 determine 17.2% of variance and represented questions about attitude towards the movement of cows; Factor 3 explained 14.1% of variance and appears to distinguish between personal opinion of handlers toward cattle.

Regarding empathy, principal component analysis identified three main factors also with eigenvalues greater than 1 (2.6, 1.7 and 1.6) that represented 71.6% of variance; Factor 1 represented 31.3% of variance representing empathy towards injured or abandoned animals; Factor 2 explained 20.9% of variance and represented questions about empathy towards pain in animals; Factor 3 explained 19.3% and represented empathy towards people or animals.

The empathy total score ranged from 20 to 100, and the attitude total score between 24 and 120. The average attitude and empathy scores for handlers were 85.05 ± 6.92 (mean ±SD; range, 73–97) and 74.61 ± 4.72 (mean ±SD; range, 65–83), respectively. Results from simple linear regression models were presented in Table 6. There was a significant difference between some handler’s interactions (prodding, shouting, raise hands and total visual interactions) during handling, in terms of the positive and negative attitude and empathy scores. Handlers with a positive attitude score used fewer prods to handle cattle, while those with a negative attitude score used more prodding. With regard to the empathy score, handlers with positive empathy scores used total visual interactions more frequently to handle cattle.

## 4. Discussion

### 4.1. Sociodemographic Characteristics and Handling Concepts

Handlers’ characteristics that may influence animal welfare standards include knowledge and being skilled at the techniques they use, job motivation and satisfaction, and attitude [3]. In this study, the handlers at the livestock market had received training in animal welfare because it is one of the pillars of the “Professional livestock handlers” training program. A high proportion of the livestock handlers interviewed were aware of the concept of the flight zone and the equilibrium point, which are necessary to achieve the movement of animals in accordance with natural behavior [2]. In addition, a high proportion of the handlers were aware of the protocol for handling non-ambulatory disabled cattle, a requirement included in the health legislation to ensure the humane handling of animals [23].

Several researchers have suggested that training sessions on the handling and management of livestock cattle has the greatest impact when implemented immediately and on a regular basis in the workplace. Practicing animal handling immediately and regularly after training and observing the consequences of their behaviors on animals’ reactions allows handlers to experience the benefits of regular training [8].

While this study did not assess the job satisfaction of livestock handlers, 44.4% of livestock handlers said that their mood was reflected in the way that they treated the cattle during the daily routine. It was recognized that the diligence with which a job is done depends very much on the level of job satisfaction [22]. However, the positive and negative emotional states of handlers can influence and motivate their behavior towards the animals; it has been suggested that positive feelings motivate behavior when there is a long-term benefit and that these emotions have reinforcing properties, and can contribute to improving the animals’ handling conditions [3]. However, given the design of this study, it would be appropriate to evaluate this aspect in more detail, in order to reach more valid conclusions.

### 4.2. Handling Assessment

Humans may unconsciously emit calming signals or ones of danger, often overlooking resultant signs of fear, aggression or calmness in the animal, and subtle differences in human behavior may be crucial [22]. In this study, negative tactile interactions used by handlers, such as hitting, prodding and tail twisting have also been the subject of frequent studies conducted on slaughterhouse livestock handlers in the United States [24] and Chile [25]. The use of negative tactile interactions can be the result of high pressure and stress exerted at the livestock market during handling. A crowded environment has a negative influence on human-animal interactions, since the livestock handler feels the need to move the animals quickly, and does not invest the time to guide the cattle carefully, which in turn results in a greater expression of fear, confusion and stress in the cattle; which is why livestock handlers will use more force and drive the animals in a more abrupt way [26]. In the present study, such interactions were significantly associated with expressions of fear, such as animals running and reversing [22,27]. In the study, behaviors considered to be abusive, such as tail twisting and the use of an electric pole (the latter not in use at the livestock market), were interactions with a very low frequency. Regarding tail twisting, it is considered to be a deeply rooted practice in the culture of cattle management in several countries such as Colombia [12,28], Chile [25] and Bangladesh [29], and several other Latin American countries [30]. The negative effect of the practice of tail twisting for cattle handling probably depends on the force used by the handler, therefore, the mere observation of this interaction does not allow one to distinguish between forceful and gentle tail twisting [31]. In this study, this practice was observed with a low frequency, because it had been discouraged and flag driving was implemented. However, these handling practices must be abandoned through the implementation and development of handling guidelines, in support of animal welfare as well as for economic reasons.

From the livestock handlers point of view, fearful animals are often more difficult to handle and manage; this, in turn, exacerbates the problems encountered during routine procedures, decreasing job satisfaction, motivation, commitment and self-esteem [32]. Cattle can also be relatively undemonstrative when hurt or severely disturbed. Human observers sometimes wrongly assume that an animal which is not squealing is not hurt or disturbed by what is being done to it. In some cases, the animal has a freezing response [33]. In this study, a high proportion of steers exhibited behavior known as freezing in the face of interactions considered as negative, such as hitting, prodding, the use of artificial noises, and shouting. Freezing is considered as a fear response by cattle, which makes them more skittish and difficult to handle [20]. In this study, auditory interactions generated limited responses in steers; similar results have been reported in cattle auctions [28]. In Colombia, auditory interactions such as shouting, which are considered negative by some authors [31], or neutral by others [34], did not affect the behavior in commercial Zebu young bulls, perhaps due to a process of habituation with this type of handling [22], taking into account that the use of human vocalizations and sounds is a common practice for the handling of commercial Zebu young bulls [12].

In this study, handlers used visual interactions such as hand raising, waving and blocking, which are consistent and accepted for minimally invasive handling of groups of cattle [2], and which are frequently used in cattle auctions in Colombia [28]. However, the study observed significant positive correlations between blocking and the behavioral responses of steers, such as slipping and falling, probably because driving represents a novel event for cattle which generates challenges, emotions and alertness [28,30].

### 4.3. Effect of the Time Elapsed within a Workday on the Handling and Behavior Reactions

Deterioration in the handling of cattle over time would be expected within a single day, as has been described in studies conducted on Brazilian cattle farms, especially among untrained livestock handlers [8]. In contrast, in the present study, handlers used more hits, prods, tail twisting and artificial sounds when handling steers at 5:00 h compared to those used at 9:00 h, which could be explained by the shorter lairage time of the cattle evaluated at 9:00 h versus those evaluated at 5:00 h (14.54 ± 1.34 h and 34.91 ± 1.70 h respectively), since the cattle handled in the first group arrived at the livestock market on Saturday, while those of the second group arrived on Sunday night or Monday morning. It is well known that long lairage time at livestock markets favor the exposure of cattle to stressors such as noise, novelty, thirst and hunger, an aspect that may have contributed to greater reactivity and difficulty during handling [10]. Studies on other species such as equines (horses, donkeys, mules and ponies) found that, once the first hour of exhibition of the specimens at a livestock market was over, they gradually displayed signs of dehydration, fatigue and compensatory metabolic problems, due to the combined effect of a number of factors, including transport and improper handling; keeping in mind that the manner of handling determines how the animals respond [9]. The reduction of positive livestock handler behavior and the increase in negative behaviors could be explained by fatigue, irritability, and poor judgement associated with the handling of exhausted animals [26], whose behavior in this study was evidenced by freezing and attempted escape.

### 4.4. Relationships between Attitude, Empathy and Human-Animal Interaction

In their work, Waiblinger et al. [22] define attitudes as the positive or negative evaluation of ‘an entity’ (species or particular animal); they are learnt through experience with or information about the animals, and they can change with new experiences or information. Thus, daily interactions may affect attitude [35]. Historically, psychologists have defined three components related to attitude: cognition, affect and conation [3]. Cognition refers to the thoughts that people have about an object; affect refers to the emotional response that a person has towards some other person or object; and conation refers to a tendency to behave in a particular way. Some authors consider that these three components are all correlated with each other and all contribute to an understanding of the underlying evaluative attitude dimension [3]. However, recent studies have suggested that this is clear evidence that reliance on the measurement of one is not sufficient to predict another [27].

In our study, handlers that generally had positive attitude and empathy scores were associated with a significant decrease in the number of negative tactile interactions, such as prodding and shouting; results that coincided with those reported by other researchers in studies on dairy farms [36,37] and extensive beef cattle systems [8]. Likewise, a study evaluating the attitudes of Japanese workers toward cows found that handlers with high positive attitude scores had a greater tendency to exhibit more positive tactile behavior (e.g., petting), and less to encourage and implement effective methods for improving attitudes, such as providing livestock handlers with new information, creating stimuli, constantly reinforcing the need to treat the animals well [8,38], and promoting actions that increase job satisfaction [39], among others.

In accordance with a psychological perspective, empathy is referred to as the capacity to understand and share the feelings of others, i.e. conspecific negative tactile behavior (e.g., hitting) when handling their animals [37]. In this respect, we observed that the livestock handlers that had negative beliefs and ideas about animals were more likely to behave negatively with them. Conversely, a livestock handler with a more positive attitude towards animals tends to exhibit less negative or aversive behavior when handling animals. In the present study, positive scores of attitude was significantly associated with an increase in the frequency of visual (hand raising) interactions of livestock handlers, which are rooted in traditional cattle management practices in Colombia [28] and are influenced by attitude [34]. It is advisable to promote the use of visual interactions such as hand raising, which are consistent with and accepted for the minimally invasive handling of groups of cattle [28]. Likewise, in order to improve the livestock handlers’ attitude towards the animals, not only is training and education sufficient and it comprises both emotional and cognitive components: cognitive empathy includes abilities such as recognizing and understanding others’ emotion self/other awareness, and perspective taking [40], whereas emotional empathy consists of the affective resonance with others’ emotions and the generation of an appropriate emotional response [7]. Some researchers have given more importance to the affective side of empathy, whereas others have taken a more cognitive approach; there is a general agreement that these two components cannot be easily separated, as empathy is a complex and multi-componential phenomenon [7].

In agricultural literature, the term ‘empathy’ has been used to describe the handler´s perceived bond with the animal under their care; likewise, degree of empathy, or some temperament factors, could predispose people to be good handlers [3]. In this research, positive scores of empathy were found in livestock handlers, and a direct relationship was found between positive scores of empathy and the frequency of total visual interactions. These are in accordance with those reported by other authors who observed that positive empathy inhibits the aggressive behavior of people [39].

The results from the current study identified associations between human-animal interactions at a livestock market level and handlers’ attitudes and empathy toward animals. Furthermore, no inferences on causality can, or should, be drawn from this work. Two limitations have been identified in this study 1) measurement biases, related to social factors, which lead to obtaining responses that are socially desirable, and 2) selection biases, due to the size of the sample and the difficulty in generalizing the results. These biases were controlled by the authors through the training of the evaluators in the use of a structured questionnaire and the evaluation of human-livestock interaction in the pilot test. The previous conditioning period of one month was used to gain the confidence of the handlers. At the beginning of the study, it was made clear to participating handlers that the results would be handled anonymously, and that they would not have any repercussions on their employment. On the other hand, the handlers who voluntarily agreed to participate in the study presented the same socio-demographic and working conditions as the cattle market handlers who did not participate in the study, an aspect which was confirmed with the database provided by the office for human resources.

## 5. Conclusions

The results of the study suggest an association between attitude and empathy towards animals with the quality of human-livestock interactions in livestock handlers under commercial conditions. Considering that livestock markets manage environments that generate stress for both people and animals, it is important to improve training, skills, handler incentives and the promotion of actions that increase job satisfaction. Good conditions should be guaranteed for the duration of the animals that stay in the livestock market facilities, with water supply, shade and food when the stay times are very long. These actions together will improve the welfare of handlers and other personnel, reduce occupational risks, and promote a good working environment. The evaluation of the attitude and empathy of handlers and the quality of their handling performance could identify poor human-animal interactions in the pre-slaughter process.

## Figures and Tables

**Table 1:** Percentages of responses (from 1 – fully disagree to 5 – fully agree) characterizing handlers (*n* = 18) attitudes toward cattle and handling behavior; and loadings of the four factors estimated by factor analysis for questionnaire data.

Attitudes	1. Fully Disagree	2	3	4	5. Fully Agree	Loadings Factor 1	Loadings Factor 2	Loadings Factor 3
It takes a good deal of physical effort to move a cow ^b^	0	11.1	5.5	38.8	44.4	−0.146	0.656	0.277
I think it is important to scratch and stroke cows ^a^	5.5	5.5	16.6	66.6	5.5	0.523	0.494	−0.250
It makes me feel good when I pet cows ^a^	0	22.2	33.3	33.3	11.1	0.780	0.138	−0.041
I remain cool and calm in excitement around me	11.1	16.6	11.1	44.4	16.6	−0.112	0.803	0.01
Cows are generally hard to handle ^a b^	11.1	27.7	5.5	38.8	16.6	−0.671	0.589	0.237
It is hard not to slap cows when moving them ^a^	22.2	44.4	0	27.7	5.5	−0.598	−0.134	0.459
Cows are dirty animals ^c^	16.6	33.3	5.5	22.2	22.2	−0.202	0.196	0.635
Talking to cows is important ^a^	5.5	5.5	38.8	38.8	11.1	0.939	−0.096	0.000
The best way to move a cow is by force ^b^	27.7	33.3	5.5	27.7	5.5	0.031	0.886	0.159
Cows are stubborn ^c^	5.5	16.6	55.5	33.3	38.8	−0.083	0.264	0.834

Questions with ^a^ represent factor loadings greater than 0.5 or −0.5 on factor 1, ^b^ represent factor loadings greater than 0.5 or −0.5 on factor 2 and ^c^ represent factor loadings greater than 0.5 or −0.5 on factor 3.

**Table 2:** Percentages of responses (from 1 – fully disagree to 5 – fully agree) characterizing handlers (*n* = 18) empathy toward cattle and handling behavior; and loadings of the four factors estimated by factor analysis for questionnaire data.

Empathy	1. Fully Disagree	2	3	4	5. Fully Agree	Loadings Factor 1	Loadings Factor 2	Loadings Factor 3
Seeing pleasant situations in animal treatment excites me ^c^	0	5.5	11.1	44.4	38.8	−0.041	−0.284	−0.720
I feel for a cow when it is injured ^a^	0	0	0	55.5	44.4	0.987	−0.074	−0.108
Occasionally I am not very sympathetic to my friends when they are depressed ^c^	5.5	27.7	33.3	27.7	5.5	−0.500	0.072	0.586
If I see that they hurt an animal I feel compassion for it. ^b^	5.5	0	0	50.0	44.4	−0.061	0.830	0.091
I am concerned about abandoned animals ^a^	0	0	11.1	44.4	44.4	0.504	0.193	−0.201
If I’m sure I’m right about something, I don’t waste much time listening to other people’s arguments. ^c^	16.6	33.3	0	27.7	22.2	−0.335	−0.045	0.799

Questions with ^a^ represent factor loadings greater than 0.5 or −0.5 on factor 1, ^b^ represent factor loadings greater than 0.5 or −0.5 on factor 2 and ^c^ represent factor loadings greater than 0.5 or −0.5 on factor 3.

**Table 3:** Adjusted means and Deviation Standard (±SD), confidential interval, number and proportion (%) of interactions of livestock handler (per min) and individual behavioral events of steers (per min) observed during handling at livestock market.

	Mean (±SD)	Confidence Interval		*n*	%
Interactions		**Min**	**Max**		
Hitting	2.67 (0.15)	2.37	2.97	484	28.78
Prodding	1.79 (0.11)	1.56	2.03	345	20.51
Prohibited practice	0.18 (0.07)	0.01	0.21	66	3.94
Shouting	1.43 (0.09)	1.24	1.62	266	15.81
Whistling	0.72 (0.07)	0.56	0.88	138	8.20
Artificial noises	0.46 (08 ± 0.92)	0.27	0.64	71	4.22
Waving	0.36 (0.05)	0.24	0.47	75	4.46
Blocking	0.12 (0.03)	0.04	0.20	24	1.42
Raise hands	1.05 (0.10)	0.84	1.27	213	12.66
Total interactions				1.682	100
Behavioral Events					
Freezing	6.39 (0.24)	5.92	6.87	1182	72.03
Reversals	0.87 (0.10)	0.67	1.07	162	9.87
Slips	0.26 (0.05)	0.15	0.37	49	2.99
Vocalizations	0.17 (0.08)	0.005	0.33	31	1.89
Falls	0.10 (0.04)	0.02	0.18	13	0.79
Running	1.05 (0.09)	0.85	1.24	204	12.43
Total behavioral events				1.641	100

**Table 4:** Pearson correlation coefficients (r) between the interactions of the handlers (per min) and behavioral events (per min) of cattle during handling at livestock market. Statistically significant correlations are presented in bold.

Interaction	Behavioral Events					
	Freezing	Running	Slips	Falls	Reversals	Vocalization
Hitting	0.53 **	0.14	−0.04	0.08	0.45 **	−0.03
Prodding	0.32 *	0.21 *	0.08	0.15	−0.02	0.04
Tail twisting	−0.06	−0.11	0.01	−0.06	0.06	0.24 **
Prohibited practice	0.01	0.08	0.11	0.07	0.05	0.2
Shouting	0.47 **	0.23 *	0.04	0.02	0.05	−0.19
Whistling	0.27 **	0.07	0.05	0.05	-	-
Artificial noises	0.33 **	0.11	0.10	−0.12	0.06	−0.12
Waving	0.07	0.01	0.02	-	−0.01	−0.05
Blocking	0.02	0.06	0.36 **	0.28 **	−0.08	0.03
Raise hands	0.18	-	−0.09	-	0.09	−0.09

* Significant correlation (*p* < 0.05) and ** (*p* < 0.01). Data log_10_(y + 1) and their square root transformed prior to statistical analysis.

**Table 5:** Effect of the time of handling within a workday (5:00, 7:00 and 9:00 h) on total livestock handlers’ interactions and total cattle behavior reactions during handling at livestock market. The duration of the handling being evaluated was included as a covariate in the analysis. The model was $P\left(k\;events\;in\;interval\right)=e^{-\lambda }\frac{\lambda^{k\;}}{k!}\;$, where λ is the average number of events per interval, e is the Euler’s number, κ takes values o, 1, 2.., κ! = κ × (κ − 1) × (κ − 2) × …× 2 × 1 is the factorial of κ.

Variable	Coefficient	SE	RR^a^ (CI)	*p* Value
Interactions				
Hitting				
5	Ref			
7	−0.11	0.10	0.9 (0.72–1.10)	0.28
9	−0.32	0.11	0.73 (0.57–0.9)	<0.01
Prodding				
5	Ref			
7	−0.05	0.12	0.95 (0.73–1.21)	0.65
9	−0.29	0.13	0.73 (0.55–0.95)	0.02
Tail twisting				
5	Ref			
7	−1.79	0.76	0.16 (0.37–0.74)	0.02
9	−0.08	0.40	1.08 (0.49–2.37)	0.8
Artificial noises				
5	Ref			
7	−0.36	0.27	0.70 (0.40–1.18)	0.18
9	−0.78	0.31	0.45 (0.25–0.84)	0.01
Behavior Reactions				
Reversals				
5	Ref			
7	−0.23	0.17	0.79 (0.55–1.12)	0.18
9	−0.70	0.2	0.49 (0.32–0.73)	< 0.01
Freezing				
5	Ref			
7	−0.08	0.06	0.92 (0.80–1.05)	0.24
9	−0.02	0.07	0.82 (0.70–0.93)	< 0.01

RR^a^ = Relative risk, SE: Standard error, RR: relative risk, CI: Confidential interval. Ref = category considered as reference.

**Table 6:** Results from simple linear regression models of the relationship between handlers’ attitude and empathy scores with the interactions used during handling: Y = β_0_ + β_1_Z_i_ + ε_i_, where Y is interactions (per min), β_0_ (intercept), β_1_ (slope), Z_i_ (predictive variable) and ε_i_ (error).

Interactions	Attitude				Empathy			
	β	Error	*p* value	R^2^	β	Error	R^2^	*p* value
Prodding (Positive attitude)	−1.37	0.54	0.02	0.28				
Prodding (Negative attitude)	3.44	1.43	0.03	0.26				
Shouting (Positive attitude)	−0.70	0.29	0.03	0.26				
Raise hands (Positive attitude)	0.93	0.36	0.02	0.28				
Total visual (Positive Empathy)					2.07	0.93	0.17	0.04

Data were transformed in their square root before the statistical analysis. R^2^ = Coefficient of determination.

## Appendix A

**Table A1:** Percentages of responses (from 1—fully disagree to 5—fully agree) characterizing handlers’ (*n* = 18) attitudes and empathy toward cattle.

Attitudes	1. Fully Disagree	2	3	4	5. Fully Agree
1.It takes a good deal of physical effort to move a cow	0	11.1	5.5	38.8	44.4
2. I think it is important to scratch and stroke cows	5.5	5.5	16.6	66.6	5.5
3. Cows react a lot to me	5.5	5.5	0	33.3	55.5
4. I think that cows can have fun	22.2	11.1	22.2	38.8	5.5
5. It makes me feel good when I pet cows	0	22.2	33.3	33.3	11.1
6. Cows are generally hard to handle	11.1	27.7	5.5	38.8	16.6
7. I remain cool and calm in excitement around me	11.1	16.6	11.1	44.4	16.6
8. It is hard not to slap cows when moving them	22.2	44.4	0	27.7	5.5
9. Cows are easy to work with	5.5	16.6	0	44.4	33.3
10. Cows respond better to shouting than to a gentle voice	50	38.8	0	11.1	0
11. Cows are dirty animals	16.6	33.3	5.5	22.2	22.2
12. Cattle are aggressive animals towards people.	22.2	16.6	11.1	27.7	22.2
13. Talking to cows is important	5.5	5.5	38.8	38.8	11.1
14. The best way to move a cow is by force	27.7	33.3	5.5	27.7	5.5
15. Cows are easily frightened	0	5.5	0	55.5	38.8
16. Cows are unable to feel happiness	16.6	33.3	22.2	27.7	0
17. Being patient with cows is very important.	0	0	0	27.7	72.2
18. Cows are stubborn	5.5	16.6	55.5	33.3	38.8
19. You need to shout at cows to make them move	0	11.1	0	22.2	66.6
20. Cows should not be rushed	5.5	11.1	5.5	50	27.7
21. Sudden movements generally scare cows	0	0	0	61.1	38.8
22. Some cows are more difficult than others	0	0	0	16.6	83,3
23.I can recognize cows individually	5.5	5.5	0	27.7	61.1
24. I treat some cows better than others	33.3	11.1	16.6	27.7	11.1
Empathy					
1. Seeing pleasant situations in animal treatment excites me	0	5.5	11.1	44.4	38.8
2. Watching videos, movies or photos of animals in certain situations makes me cry.	5.5	22.2	16.6	50.0	5.5
3. Work does not give me time to put myself in the other’s place or to reflect to make decisions.	11.1	27.7	5.5	50.0	5.5
4. I feel for a cow when it is injured	0	0	0	55.5	44.4
5. Occasionally I am not very sympathetic to my friends when they are depressed.	5.5	27.7	33.3	27.7	5.5
6. Sick animals worry me	0	0	5.5	61.1	33.3
7. When a friend tells me about his good fortune, I feel genuinely happy for him	0	0	0	44.4	55.5
8. If I see that they hurt an animal I feel compassion for it.	5.5	0	0	50.0	44.4
9. I think that every situation has several possibilities of solution.	0	11.1	0	38.8	50.0
10. I care for my friends a great deal.	5.5	0	11.1	61.1	22.2
11. I am concerned about abandoned animals.	0	0	11.1	44.4	44.4
12. When I see an injured cow, I feel like helping it.	0	0	5.5	38.8	55.5
13. If I’m sure I’m right about something, I don’t waste much time listening to other people’s arguments.	16.6	33.3	0	27.7	22.2
14. Seeing a neglected steer doesn’t affect me as much as it would affect most people.	5.5	11.1	22.2	55.5	5.5
15. I do not find calves cute	44.4	22.2	5.5	16.6	11.1
16. I try to understand cows by imagining how things look from their point of view	0	0	11.1	66.6	22.2
17. Imagining how a cow feels is something I could not easily do	55.5	11.1	0	11.1	27.7
18. When I see an unhappy cow it upsets me more than it would most people	0	5.5	27.7	50.0	16.6
19. I get very upset when I see a cow in pain.	0	0	16.6	55.5	27.7
20. Before scolding an animal, I try to imagine how I would feel if I were in its place.	0	0	77.7	11.1	0

## References

[1] Boissy A., Manteuffel G., Jensen M.B., Moe R.O., Spruijt B., Keeling L.J., Winckler C., Forkman B., Dimitrov I., Langbein J. (2007). Assessment of positive emotions in animals to improve their welfare. Physiol. Behav..

[2] Grandin T., Grandin T. (2015). The effect of economic factors on the welfare of livestock and poultry. Improving Animal Welfare: A Practical Approach.

[3] Hemsworth P., Coleman G., Hemsworth P., Coleman G. (2011). Human-Livestock Interactions: The Stockperson and the Productivity and Welfare of Intensively Farmed Animals.

[4] Coleman G., Hemsworth P., Hay M., Cox M. (2000). Modifying stockperson attitudes and behaviour towards pigs at a large commercial farm. Appl. Anim. Behav. Sci..

[5] Pinillos R.G., Appleby M.C., Manteca X., Scott-Park F., Smith C., Velarde A. (2016). One Welfare—A platform for improving human and animal welfare. Vet. Rec..

[6] Losada-Espinosa N., Miranda-De la Lama G.C., Estévez-Moreno L.X. (2020). Stockpeople and Animal Welfare: Compatibilities, Contradictions, and Unresolved Ethical Dilemmas. J. Agric. Environ. Ethics.

[7] Colombo E.S., Crippa F., Calderari T., Prato-Previde E. (2017). Empathy toward animals and people: The role of gender and length of service in a sample of Italian veterinarians. J. Vet. Behav..

[8] Ceballos M.C., Sant’Anna A.C., Boivin X., de Costa F.O., de Carvalhal M.V.L., Paranhos da Costa M.J.R. (2018). Impact of good practices of handling training on beef cattle welfare and stockpeople attitudes and behaviors. Livest Sci..

[9] Corrales-Hernández A., Mota-Rojas D., Guerrero-Legarreta I., Roldan-Santiago P., Rodríguez-Salinas S., Yáñez-Pizaña A., de la Cruz L., González-Lozano M., Mora-Medina P. (2018). Physiological responses in horses, donkeys and mules sold at livestock markets. Int. J. Vet. Sci. Med..

[10] Stojkov J., von Keyserlingk M.A.G., Duffield T., Fraser D. (2020). Fitness for transport of cull dairy cows at livestock markets. J. Dairy Sci..

[11] Destrez A., Haslin E., Boivin X. (2018). What stockperson behavior during weighing reveals about the relationship between humans and suckling beef cattle: A preliminary study. Appl. Anim. Behav. Sci..

[12] Romero M.H., Uribe-Velásquez L.F., Sánchez J.A., Rayas-Amor A.A., Miranda-de la Lama G.C. (2017). Conventional versus modern abattoirs in Colombia: Impacts on welfare indicators and risk factors for high muscle pH in commercial Zebu young bulls. Meat Sci..

[13] Instituto Colombiano Agropecuario Reglamento que establece las condiciones sanitarias y de inocuidad de la producción primaria de ganado bovino y bufalino destinado al sacrificio para consumo humano. https://www.ica.gov.co/getdoc/016f3c96-a458-4fa6-ae96-41d18b2221f5/requisitos-sanitarios-y-de-inocuidad-en-la-producc.aspx..

[14] Boivin X., Marcantognini L., Boulesteix P., Godet J., Brulé A., Veissier I. (2007). Attitudes of farmers towards Limousin cattle and their handling. Anim. Welf..

[15] Hanna D., Sneddon I.A., Beattie V.E. (2009). The relationship between the stockperson’s personality and attitudes and the productivity of dairy cows. Animal.

[16] Norring M., Wikman I., Hokkanen A.-H., Kujala M.V., Hänninen L. (2014). Empathic veterinarians score cattle pain higher. Vet. J..

[17] World Health organization Process of translation and adaptation of instruments. https://www.who.int/substance_abuse/research_tools/translation/en/.

[18] Grandin T., Grandin T. (2007). Behavioural principles of handling cattle and other grazing animals under extensive conditions. Livestock Handling and Transport.

[19] Breuer K., Hemsworth P., Barnett J., Matthews L., Coleman G. (2000). Behavioural response to humans and the productivity of commercial dairy cows. Appl. Anim. Behav. Sci..

[20] Lindahl C., Pinzke S., Herlin A., Keeling L.J. (2016). Human-animal interactions and safety during dairy cattle handling—Comparing moving cows to milking and hoof trimming. J. Dairy Sci..

[21] Hemsworth P. (2003). Human–animal interactions in livestock production. Appl. Anim. Behav. Sci..

[22] Waiblinger S., Boivin X., Pedersen V., Tosi M.-V., Janczak A.M., Visser E.K., Jones R.B. (2006). Assessing the human–animal relationship in farmed species: A critical review. Appl. Anim. Behav. Sci..

[23] Instituto Colombiano Agropecuario I. Reglamento que establece los requisitos para la expedición de licencias zoosanitarias de funcionamiento que autorizan las concentraciones de animiales y se señalan los requisitos sanitarios para los animales que participen en ellas. https://www.ica.gov.co/getdoc/016f3c96-a458-4fa6-ae96-41d18b2221f5/requisitos-sanitarios-y-de-inocuidad-en-la-producc.aspx.

[24] Grandin T. Restaurant Animal Welfare and Humane Slaughter Audits in U.S.. http://www.grandin.com/_.

[25] Strappini A.C., Metz J.H.M., Gallo C., Frankena K., Vargas R., de Freslon I., Kemp B. (2013). Bruises in culled cows: When, where and how are they inflicted?. Animal.

[26] Grandin T. Recommended Animal Handling Guidelines & Audit Guide (2005 Edition With 2007 and 2010 Updates). http://animalhandling.org/sites/default/files/forms/animal-handling-guidelines-.

[27] Hemsworth P.H., Rice M., Karlen M.G., Calleja L., Barnett J.L., Nash J., Coleman G.J. (2011). Human–animal interactions at abattoirs: Relationships between handling and animal stress in sheep and cattle. Appl. Anim. Behav. Sci..

[28] Herrán L., Romero M., Herrán L. (2017). Interacción Humano-Animal y Prácticas de Manejo Bovino en Subastas Colombianas. Revista de Investigaciones Veterinarias del Perú.

[29] Alam M.R., Gregory N.G., Jabbar M.A., Uddin M.S., Kibria A.S.M.G., Silva-Fletcher A. (2010). Skin injuries identified in cattle and water buffaloes at livestock markets in Bangladesh. Vet. Rec..

[30] Roldan P., de la Cruz L., Tarazona A., Buenhombre J., Acerbi R., Varona E., Mota D., Mota-Rojas D., Velarde A., Huertas S.M., Cajiao M. (2016). Bienestar animal en mercados ganaderos. BIENESTAR ANIMAL, una visión global en Iberoamérica.

[31] Pajor E., Rushen J., de Passillé A.M. (2000). Aversion learning techniques to evaluate dairy cattle handling practices. Appl. Anim. Behav. Sci..

[32] Waiblinger S., Menke C., Coleman G. (2002). The relationship between attitudes, personal characteristics and behaviour of stockpeople and subsequent behaviour and production of dairy cows. Appl. Anim. Behav. Sci..

[33] Broom D., Grandin T. (2007). Causes of poor welfare and welfare assessment during handling and transport. Livestock Handling and Transport.

[34] Breuer K., Hemsworth P., Coleman G. (2003). The effect of positive or negative handling on the behavioural and physiological responses of nonlactating heifers. Appl. Anim. Behav. Sci..

[35] Lürzel S., Barth K., Windschnurer I., Futschik A., Waiblinger S. (2018). The influence of gentle interactions with an experimenter during milking on dairy cows’ avoidance distance and milk yield, flow and composition. Animal.

[36] Sorge U.S., Cherry C., Bender J.B. (2014). Perception of the importance of human-animal interactions on cattle flow and worker safety on Minnesota dairy farms. J. Dairy Sci..

[37] Fukasawa M., Kawahata M., Higashiyama Y., Komatsu T. (2017). Relationship between the stockperson’s attitudes and dairy productivity in Japan. Anim. Sci. J..

[38] Pulido M.A., Mariezcurrena-Berasain M.A., Sepúlveda W., Rayas-Amor A.A., Salem A.Z.M., Miranda-de la Lama G.C. (2018). Hauliers’ perceptions and attitudes towards farm animal welfare could influence the operational and logistics practices in sheep transport. J. Vet. Behav..

[39] Adler F., Christley R., Campe A. (2019). Invited review: Examining farmers’ personalities and attitudes as possible risk factors for dairy cattle health, welfare, productivity, and farm management: A systematic scoping review. J. Dairy Sci..

[40] Signal T.D., Taylor N. (2007). Attitude to Animals and Empathy: Comparing Animal Protection and General Community Samples. Anthrozoos.

